# Healthy Dietary Choices and Physical Activity Participation in the Canadian Arctic: Understanding Nunavut Inuit Perspectives on the Barriers and Enablers

**DOI:** 10.3390/ijerph18030940

**Published:** 2021-01-22

**Authors:** Victor O. Akande, Timothy O. Fawehinmi, Robert A.C. Ruiter, Stef P.J. Kremers

**Affiliations:** 1 Department of Health Promotion, NUTRIM School of Nutrition and Translational Research in Metabolism, Maastricht University Medical Center+, Maastricht University, P.O. Box 616, 6200 MD Maastricht, The Netherlands; s.kremers@maastrichtuniversity.nl; 2 Department of Health, Government of Nunavut, Iqaluit, NU X0A 0H0, Canada; dapofawehinmi@gmail.com; 3 Department of Work & Social Psychology, Faculty of Psychology & Neuroscience, Maastricht University, P.O. Box 616, 6200 MD Maastricht, The Netherlands; r.ruiter@maastrichtuniversity.nl

**Keywords:** healthy, diet, activity, behavior, Inuit, perspectives, photo-elicitation, cost, affordability, resources

## Abstract

Background: Research shows that unhealthy diets and low physical activity are associated with high rates of obesity-linked chronic diseases amongst Nunavut Inuit. To provide contextual insights and deepen our understanding of the factors that underlie these lifestyle choices, we explored the perspectives of Nunavut Inuit on the barriers and enablers of healthy diets and physical activity participation in the community of Iqaluit. Methods: One-on-one semi-structured photo-elicitation interviews were conducted with 16 participants of 18 years and over (10 women, six men). The interviews uncovered the participants’ perspectives on the factors influencing healthy diets and physical activity in their community. Interviews were audio-recorded, transcribed, and uploaded to QSR NVIVO Version 12. Data analysis was achieved using an inductive thematic approach. Results: Six main factors were identified as barriers or enablers to energy balance-related behaviors: cost and affordability of healthy choices; availability of traditional foods and activities; weather conditions and climate change; infrastructure and community resources; social networks of family and friends; and effect of substance use. Conclusion: This study identified six broad areas that should be considered while mapping out interventions to reduce the burden of obesity-related chronic diseases in Nunavut communities.

## 1. Introduction

Inuit diets and activity patterns have significantly changed over the past five decades in response to the socio-cultural and environmental transitions in the Arctic. The population has transitioned from the traditional hunter-gatherer subsistence living to Euro-Canadian ways of life. This lifestyle transition is characterized by steady increases in the consumption of energy-dense processed foods [1,2,3] and diminishing rates of physical activity and fitness level [4,5,6,7].

Although the Inuit population is growing at a rate higher than the national average [8], many Inuit continue to live in communities with limited access to healthy food supplies [6] and poor infrastructure for recreational activities. The social conditions are generally suboptimal and appear to be a major contributor to the rise in diet-related health problems such as obesity, and chronic diseases such as type 2 diabetes [9,10]. For example, type 2 diabetes was alien to the Inuit population five decades ago, but the disease is present nowadays at a rate that is similar to or higher than the mainstream Canadian population [11].

A body of research evidence has demonstrated comparative health, social, and economic benefits of sustained harvesting of traditional foods in the Canadian Arctic over energy-dense store-bought processed foods [12,13,14]. However, healthy traditional foods are consumed less. In their cross-sectional study in Nunavut, Sharma and colleagues found that consumptions of essential vitamins and fibers were lower than recommendations, and significantly less traditional foods were consumed in contrast to sugar-sweetened beverages and energy-dense processed foods [15]. An age-dependent generational shift from traditional food consumption by older Inuit to store-bought processed foods by the younger Inuit population has been described [16,17,18,19]. This change is equally evident in the occupational patterns and the switch from hunting and subsistence living to a wage economy [18,19] characterized by limited physical activity and associated energy expenditure. Younger Inuit men and women are less likely to participate in hunting activities, as they generally lack the knowledge and experience compared to their parents and grandparents [20]. As the Inuit population has transitioned from nomadic living to more permanent settlements, food-gathering activities, including hunting, fishing, and trapping, nowadays require the use of motorized equipment [19] such as snow machines, four-wheeled all-terrain vehicles, and boats, as well as firearms. Moreover, a hunter’s success largely depends on his disposable income; that is, the ability to afford the high cost of hunting equipment, including fuel. Fewer hunting and food gathering activities also means decreased opportunities for physical activity and increased consumption of energy-dense store-bought foods [6]. All these developments are suggestive of less physical activity within an increasing obesogenic environment, and they are likely to combine to increase obesity rates and chronic diseases in the population.

To date, there have been limited studies that aimed at advancing our understanding of the determinants of diet and physical activity among Inuit [6,7]. Notably, most previous studies had quantitative research designs, which limits our understanding of belief structures and underlying contexts. We have previously shown in a systematic review [6] that qualitative studies that directly examined the perspectives of Nunavut Inuit on factors influencing their dietary and physical activity patterns are scarce. The main findings from the review indicated that sociocultural and environmental changes are responsible for transitions from healthy traditional diets to lower-nutrient, energy-dense store-bought foods in Inuit communities. We also documented a shift in lifestyle among Inuit from an active hunter-gatherer subsistence living to a more sedentary and motorized lifestyle over the last 50 years [6]. A qualitative inquiry may provide contextual insights and deepen our understanding of the underlying factors that embed the dietary choices and physical-activity participation in the population. Low literacy levels, socioeconomic status of research participants, and cultural differences between research participants and the researcher often limit the ability of the researcher to gain insights into the world of research participants. Thus, it becomes challenging to engage in discourses and capture salient information that cannot be provided through the use of questionnaires. It is necessary to understand the perspectives of Inuit on the factors that constitute barriers and enablers to making healthy dietary choices and increasing the physical activity levels in the population. An in-depth understanding of the factors that is predicated on Inuit’s lived experiences is critical to developing appropriate interventions by policymakers and public-health officers.

In line with the proposed research objectives in this Indigenous population, we employed a photo-elicitation method for this qualitative inquiry. According to Harper [21], photo-elicitation is a research approach that bridges the cultural gap between the researcher and the participants. While integrating photographs into their research, Epstein and colleagues described photo insertion in the research method as “ice breaker activity to create a comfortable space for discussion between two individuals who perhaps have different cultural identities and hold differing world views” [22]. This approach is particularly relevant given the cultural differences between researchers and the Inuit research participants. In the current study, photo-elicitation was used to initiate conversation on the research topics, and through participants’ lived experiences and views, uncover the barriers and enablers of healthy dietary choices and physical-activity participation.

## 2. Materials and Methods

This study was part of a larger project that aimed to explore the determinants of dietary behavior and physical activity in the Canadian Arctic. The Inuit population in Nunavut and the nature of the built and food environments have been previously described [6,7]. We utilized an exploratory qualitative study approach to understand the barriers to and facilitators of healthy dietary choices and physical-activity participation among Inuit. Approvals for the study were obtained from the ethics review committee for psychology and neuroscience at Maastricht University, The Netherlands (reference number ECP-148 05_03_2015), and the Nunavut Research Institute (License number 050 1315-Amended).

### 2.1. Study Population

The Inuit are one of the three Indigenous peoples of Canada [23]. The Inuit population has the highest growth rate and is the youngest demographic group in Canada, with a median age of 22, compared to 40 nationally [24]. Participants in the qualitative study were recruited in Iqaluit, the territorial capital. Iqaluit, which is the largest and the most culturally diverse community in Nunavut, has a population of 7950, of whom 55.4% are Inuit and 44.6% are non-Inuit. Participants were Nunavut Inuit that were 18 years or older and had lived in Nunavut for at least 10 years prior to the study, and were fluent in the English language.

### 2.2. Photo-Elicitation Interviews

Data collection was divided into two phases. Phase one consisted of photoshoots in the community and collection of historical photographs for the photo elicitation interview (PEI). PEI is defined as the insertion of photographs in an interview. Photographs are used in qualitative inquiry to trigger memory and evoke responses to interview questions. In the current study, we included photographs in the semi-structured interviews that were conducted with Inuit participants.

Two interviewers, VOA and TOF, took photographs of the contemporary built and food environments in Iqaluit during the summer and winter, and collected a few historical photographs of the neighborhoods, streets, houses, motor vehicles, walkways, playgrounds, fitness/sports center, etc. The historical photographs depicted the community built and food environments as they were 30 to 50 years ago. The contemporary photos included photographs of food items in the two main grocery stores with prices, as well as traditional foods, to gather photographic representations of the current food environment. These photographs were then included in the photo elicitation interviews to advance our understanding of how certain components of the food environment might affect dietary choices for Iqaluit residents. In addition, photographs of roads, streets, walkways, parks, and the sport/fitness center, as well as objects such as buildings, motor vehicles, snow mobiles, etc., were taken to capture the current state of the built environment in Iqaluit. Photograph selection for the interviews (both contemporary and historical) was guided by the perceived environmental attributes in the built environment and known to influence walking behavior [25,26]. The aim was to understand, from the perspective of Iqaluit residents, how neighborhood attributes such as the constructs of land-use mix diversity and land-use mix access, perceived safety from traffic and crime, and perceived levels of infrastructure and facilities for walking, cycling, and neighborhood aesthetics, constitute barriers or enablers of physical activity. Ten photographs (five historical and five contemporary) were selected for each of the built and food environments. It is worthy to note that the interviewers VOA and TOF are not necessarily outside researchers who “parachuted” into the territory; rather, they are Nunavut residents with a reasonable understanding of the Inuit culture and ways of life. They have both lived in communities in the territory over a period of six to eight years, where they worked with Inuit families and community leaders.

Participants were recruited using a purposeful sampling technique [27]. The sample size was guided by the recommendations of Clarke and Braun [28] and Fugard and Potts, [29] for data and thematic saturations. Posters were advertised in public areas such as grocery stores and in the community center. Information sheets describing the study were provided to potential participants who initially expressed interest in the study. The information was written in plain language at a reading-grade level of six, and contained information such as the participation criteria, opt-in and opt-out procedures, confidentiality requirements, ownership, control, and access to data. In total, 22 participants (14 women and 8 men) were initially recruited for this study upon showing interest, and demonstrating an understanding of the research goal and participation process through a question-and-answer session. These individuals were fully briefed in two separate sessions about the purpose of the study and, finally, the signing of the research consent form. Of the 22 who initially expressed interest, 16 respondents (10 women and 6 men) were included in semi-structured photo-elicitation interviews and subsequent data analysis. Of the six participants who dropped out of the study, three persons (two female, one male) informed the researcher of their inability to participate due to family commitments. The remaining three persons (two female and one male) expressed a desire to drop out due to a lack of interest in the areas of research focus.

One-on-one semi-structured interviews were conducted based on the photographs. Each participant was asked to select five photos from the 10 photographs that were presented by the researcher for discussions of food and built environments. Each participant chose five photographs of interest from a list of healthy traditional and non-traditional foods, including caribou meat, seal meat, arctic char, pasteurized milk, berries, spinach, etc.; and unhealthy foods such as potato chips, frozen pizza, white bread, cookies, sugar-sweetened beverages, etc. Healthy and unhealthy diets amongst Nunavut Inuit were previously described in detail in a systematic review [6]. Five photographs of interest were also chosen by each participant to elicit conversation about the built environment and infrastructure in the city of Iqaluit in relation to physical-activity participation. These included the streets, walkways, sports center, buildings, park, motor vehicles, snowmobiles, etc. For each group of five photos, semi-structured questions were asked. Participants were first asked to describe each photo, followed by what they perceived as the event that was captured in the picture and how this related to food choices and consumption pattern of Inuit and Inuit life generally. Further, participants described what factors or elements constituted barriers or facilitators of a healthy diet, both personal and structural, in the past and in the contemporary age. Similar questions for the built environment and physical activity were asked, including what factors or elements based on what they saw in the photos constituted barriers or facilitators of an active living lifestyle in their community. Interviews were successfully conducted with 16 participants, although data saturation was reached after interviewing the 14th participant. At this point no new information was provided by research participants and no additional themes were generated on the topics under investigation. Interviews were audio-recorded and later transcribed using Rev Transcription Software 2017, San Francisco, CA, USA.

Data analysis was conducted using an inductive thematic approach [30]. By way of confirmation, the themes, subthemes, and insights generated from the data were member-checked by the participants [31]. Following data collection and analyses, VOA and TOF returned to research participants to briefly present, and confirm, if the findings and interpretations reflected participants’ realities, and participants were in agreement with the conclusions reached based on their own individual interviews. Participants were largely in agreement except for minor corrections that yielded additional information during clarification.

Transcribed data were uploaded to QSR NVIVO Version 12 to conduct text searches and word-frequency queries, find connections within data, and generate patterns and themes. We utilized a three-stage coding system. In the first stage, each transcript was read repeatedly to identify phrases that could be organized into general themes. In the second stage (axial/pattern coding), the initial codes that were developed in stage one were grouped into smaller themes. In stage three (selective coding), all codes were reviewed and further refined into thematic categories. The second coder, TOF, cross-checked the three coding stages, and based on discussion with VOA, made changes to the codes with mutual agreement. The thematic categories were then linked through logical deductions to achieve the highest level of validation for the central themes and subthemes presented below. In the Results section, it is stated whether the participant was male (M) or female (F).

## 3. Results

The factors hindering and facilitating physical-activity participation and healthy eating among Canadian Inuit from participants’ perspectives are categorized under six broad themes: cost and affordability of healthy choices; availability of traditional foods and activities; weather conditions and climate change; infrastructure and community resources; social networks of family and friends; and effect of substance use.

### 3.1. Cost and Affordability of Healthy Choices

The main perceived barrier to healthy food choices and participation in physical activity that participants identified was affordability. Participants mentioned that many community residents cannot afford the high costs of healthy foods, both traditional (e.g., caribou meat, seal meat, arctic char, etc.), and non-traditional foods such as beef, milk, eggs, fruits, and vegetables, which could be 50% to 300% higher than the prices in the southern part of Canada. They noted that unhealthy (junk) foods are cheaper and preferred by many Inuit with low income. According to the participants, traditional foods are increasingly becoming expensive because of the cost of hunting equipment and lower availability of hunters with the appropriate hunting skills:

*“If you consider everything, it will drive you crazy, so you have to stop looking at the prices… … Every now and then I’ll look at the price, but not very often because it just drives you crazy. Although, I get to the point when I’m really angry because I know I don’t need to pay this much, you know? So you kind of walk over … You’re not angry at anybody, you’re just kind of angry that you have to pay this much”.*

(F)

*“I believe they’re the ones that are in control of the food prices for many of the healthy foods. If there were other ways to bring it up north, more in bulk, and cheaper ways, I think food prices would be lower cost, they’re the ones controlling it all I believe”.*

(M)

*“Especially having lived elsewhere where the food was affordable, then it’s great, you know. But if you haven’t gone anywhere then they don’t know any better, you know? We ordered a club sandwich from the snack, it cost us $50. She actually posted it and said, “Look, a $50 club sandwich”.*

(F)

*“…and ordered breakfast and paid a $30 price for breakfast, which would only cost $7 in Ottawa, you know?”.*

(F)

Cost and affordability impact physical activity as well. The high cost of hunting equipment, resulting in less hunting activities, and the exorbitant cost of registration fees at fitness centers or sports equipment in the face of relatively low household income, all combine to reduce physical-activity rates and fitness levels in the Inuit population:

*”You know, there’s a difference. Not every family, in a family has machines to go hunting. When we all had dogsleds, everybody went, everybody could go, but now we have machines to take us everywhere, and none of them are under 10 grand”.*

(F)

*“You’ve got to be rich to play [ice] hockey. So you’ve got to make sports accessible to Inuit and not charge them a lot because it’s them that suffer when you do a bit pull like that, do you know what I mean? It’s only mostly the elite which are all from the southern part. So hockey maybe not be a good example to show me”.*

(F)

### 3.2. Availability of Traditional Foods and Activities

Another barrier that was mentioned by participants was related to availability of healthy choices including traditional foods and cultural activities. Some participants argued that it is one thing to know the importance of healthy food choices and be interested in adopting healthy eating as a lifestyle, and be able to afford this, while it is a different issue entirely to find these healthy food options in the store on a regular basis. Explaining further, some respondents commented that there are certain government regulations that restrict the number of animals such as caribou that can be harvested in certain parts of the territory, thus impacting its availability and accessibility.

*“[Traditional food] is accessible to certain degree. For example, we have the country food shop near the library, which is nice. We can go there and grab tuktu, grab fish, but sometimes there’s also moments where you’ll go looking for something and you can’t find that. That’s one. Two, there’s also like the days where you can’t really go hunting because of the weather, because nowadays the weather is very topsy turvy. You can’t predict it as much so I’d say those are the two main factors”.*

(F)

*“For caribou when my nephews and cousins travel to the west, where they have no quota limit on caribou, if it’s available and they are coming back with fresh caribou. If family or friends share their, what they have, if it’s family dinner going on”.*

(M)

Several decades ago, traditional hunting was a lifestyle and a cultural activity that promoted active living and provided healthy food supplies to households. Inuit lived a nomadic and active lifestyle, promoting physical health and fitness. One participant stated:

*“I think back then it was a lot more physical activity because we were, and what I know we were often migrating with the animals, following their migration routes. Moving from camp to camp, consistently looking for food and maintaining shelters, and clothing, hunting tools. I believe one was never idle back then, I think idle back then was considered lazy and I believe today there’s a lot more, more things are given to us, and we are taking it for granted. That they’re able to, not required to do as much to live in today’s age”.*

(M)

According to some participants, a general shift from traditional hunting as a means of subsistence to a wage economy also influences availability:

*“So not everybody can do that. Most of our people are working nine to five, Monday to Friday, so they don’t do this on a regular basis, but they may go out and do a variation of this on the weekend”.*

(F)

Moreover, colonization and sociocultural assimilation have disrupted Inuit cultural practices and disparaged traditional values, such that many Inuit have attached less social significance to hunting and as a way of life, and suggesting that “food sharing” practices should be promoted as traditional value to improve access.

*“But we’re totally immersed in this [Euro-Canadian] culture here in….. not so much in the communities”*

*“I get access to … If I had relatives that went hunting, people share. They’re on Facebook, people saying, “We’re having the meal today, please come. Anybody come.” Those are the other options. The third option, final option, would be to go to a store. We have a country food store here. It’s an arm and a leg, but when we’re craving we crave big”.*

(F)

### 3.3. Weather Conditions and Climate Change

The changing climatic conditions were identified by some participants as contributing to low participation in activities that are tied to healthy diets and active living. As well, it was suggested that the long winter season that ranges from six to eight months at very low temperatures are deterrent to engagement in physical activity, thus reducing participation rates.

Commenting on the effect of weather on physical activity, a participant opined:

*“Sometimes it’s cold outside. You don’t wanna walk all the way……so you just decide not to go, you know? But yeah, also the weather conditions. You know that a lot of people don’t even like getting out of their house during the winter ‘cause it’s so dark out, you know, so mentally they just kind of don’t wanna do that stuff anymore”.*

When asked of one perceived barrier to active living as a lifestyle, one participant said:

*“The cold weather. If it’s very cold out, I try and stay indoors”.*

(M)

According to one participant:

*“[The government] is not willing to acknowledge that climate change and global warming, are occurring and having a large impact on the circumpolar nations…..and [Inuit] culture will slowly diminish away, where they will not be able to practice their traditional practices of hunting on their land. Because if global warming continues, then [this] particular ice flow, this ice rift that’s here, will become much wider apart, and it’ll be far unsafe for this particular person to go out on the land to hunt for any sea animals”.*

(F)

Further into the discussion on weather-related challenges that Inuit face while trying to access resources during winter, one participant emphasized the need for adequate facilities and the need for these amenities to be strategically located in the community. She argued:

*“…but some people who live, for example, all the way in Happy Valley, or not even Happy Valley, in like Tundra Valley or something. They’re gonna probably be less likely to drive all the way, especially in the winters, drive all the way to work and they drive all the way back. It’s not something that a lot of people do, so just having the facilities open, whether that’s closer to a lot of household places, in buildings, stuff like that”.*

(F)

### 3.4. Infrastructure and Community Resources

Nunavut is a resource-limited environment compared to southern provinces. The availability of adequate resources and the level of infrastructural development in Inuit communities continue to pose barriers to healthy diets and active living, according to respondents. Thus, increasing community-level resources would increase the participation rates in physical activity and the chances that a healthy eating behavior would be adopted in the population:

*“…We don’t have sidewalks here in Iqaluit. We have no sidewalks, so we walk on pavement. So to have sidewalks where people could actually walk on. A place where you can put salt on to de-freeze things, you know, preventing you from slips, of black ice, where most people can actually walk, because if you were to have those things set up, then perhaps people would want to engage in more activities outdoors”.*

(F)

*“…in the winter the sidewalks are not as maintained. In the summer you could sort of go anywhere, and everywhere quicker as compared to walking in deep snow, or snow banks to go around”.*

(M)

Commenting further, a participant commented on the need to engage community elders as resource persons to teach Inuit traditions and transfer cultural legacies such as hunting to increase the number of Inuit who are accessing healthy traditional foods:

*“… And…[give] opportunities to children and adolescence to learn the skills of hunting. Because it’s important to continue the traditions onward as opposed to it simply dying from the elders and not being passed through the intergenerational learning”.*

(F)

Although resources and infrastructures are limited, adequate funding and personnel continue to pose significant challenges to the maintenance of existing infrastructures in the community:

*“Hmm. It exists, at least from my understanding, it exists from the infrastructure that’s present within the north. It’s quite difficult to maintain sidewalks, because realistically, we are living north of 60. We are in a circumpolar area, and that results in lots of snow falling. And the maintenance of infrastructure such as sidewalks is uncertain, because I’ve seen before that there are sidewalks that were created by putting large rocks on the side of the road. And this creates a sidewalk, giving that illusion that it is that. But at the same time, in order for it to change, it’s difficult, because you can’t do so much with the municipal budget to create a sidewalk that will eventually get covered up by snow”.*

(F)

### 3.5. Social Networks of Family and Friends

Healthy choices tend to converge with those of close social connections, including family and friends, especially in settings where communal lifestyle and social inclusion and cohesiveness are important cultural values. Norms of appropriate food choices and participation in physical activity are set, or at least influenced, by the behaviors of other people, but also by shared cultural expectations and environmental cues. Participants indicated that Inuit are likely to follow choices that are perceived “acceptable” based on social comparison:

*“To keep healthy, but also my parents tease me a lot so I think that’s another reason why I work out. ‘Cause I came back from international travel. I went to Africa for a couple of weeks, and so I ate a lot of rice there. My parents came up and they saw me and they’re like, “…, I think you’ve gained some weight.” They don’t bother me about it. They just kind of mentioned it, but I’m like, hey, and I also feel like my pants are a bit tighter now. So I’m, “Okay, I need to actually buckle up and to get a bit healthier.” Also you feel good too, so …yeah”.*

(F)

*“Primarily just my family household. My dad works out, my brother works out. We go hiking and stuff like that. That’s one thing, but other things? If I was to be totally honest with you I think a lot of it is also just how people … You know, like fat shaming, stuff like that…”.*

(F)

*“Social things like how, if you’re fat you’re “fat,” in quotation marks. If you’re fat then you’re just less of a human being. So I think there’s a lot of social stigma that goes around it. For me though, specifically, I think it’s just wanting to look healthy and fit for myself”.*

(F)

### 3.6. Effects of Substance Use

Further, participants were asked about other healthy and unhealthy habits they engaged in, and whether any of these played a role in healthy eating behavior and physical activity participation. Engagement in certain unhealthy behaviors was a barrier to participation in physical activity and healthy diet choices for many of the participants and relatives. One participant said:

*“Because I smoke cigarettes all the time and it makes me tired if I try to exercise”.*

(M)

A packet of cigarette costs $20 to $25 in Nunavut, and on average a smoker consumes about 10 sticks per day. Alcohol consumption is significantly high, even in communities where alcohol is prohibited. A bottle of liquor is sold for over $200. In many households, cigarette/marijuana smoking, drug use, and alcohol consumption all combine to decrease the available funds to purchase healthy foods, ice-hockey equipment, and gym memberships. On why a lot of people do not exercise, one participant responded:

*“Smoking weed? Drinking booze? There’s too many… that drink and smoke weed, so it affects their kids too. So yeah, it affects a lot of kids”.*

(M)

Another participant corroborated the influence of these unhealthy behaviors:

*“It affects us a lot, especially for us people that have no employment. That are seeking like … drug dealing or bootlegging, they resort to that, because they don’t have money to feed their children or to put food on the table. So they have to you know, make … find another way to make money you know”.*

(M)

Some respondents emphasized the need to promote Inuit health through public education, ensuring that people understand the benefits of quitting binge drinking, smoking and drug use, and promoting the adoption of healthy behaviors such as healthy food choices and active living as a living lifestyle:

*“We need to tell people to quit smoking, to find a new habit, we need therapy … we need a lot of therapy for our cigarettes”.*

(M)

## 4. Discussion

The aim of this study was to explore the barriers and facilitators of healthy diet and physical-activity participation among Canadian Inuit adults in Nunavut. Findings from participant interviews and data analyses indicated that six factors, acting either as barriers or facilitators, are essential to the promotion of healthy eating and physical activity promotion amongst Nunavut Inuit: cost and affordability of healthy choices; availability of traditional foods and activities; weather conditions and climate change; infrastructure and community resources; social networks of family and friends; and effects of substance use.

The most significant perceived barrier to a healthy energy-balance-related behavior is the cost and affordability factor. This is largely due to low incomes and high levels of poverty amongst Nunavut Inuit, and is further aggravated by the exorbitant costs of both traditional and non-traditional foods. In addition, the high costs of hunting equipment, resulting in fewer hunting activities, and the exorbitant cost of registration fees at fitness centers or sports equipment, in the face of relatively low household income, all combine to reduce physical-activity rates and fitness levels in the population. Our findings are corroborated by a body of research evidence [32,33,34,35] that demonstrated an influencing role of income on the consumption patterns of the Canadian Inuit. For example, Hopping and colleagues [33] established a positive correlation between income and frequency of fruit and vegetable consumption in Nunavut. In response to the cost and affordability-related persistent food-insecurity issues in Nunavut, community leaders and public health advocates have relentlessly asked the federal and territorial governments for intervention programs. Nutrition North Canada, a food subsidy program that was launched in 2011, was the federal government’s direct response to the call [36]. The subsidy focuses on perishable healthy foods, and the fund is administered by southern suppliers and local retailers in the community, who then pass on the subsidy to consumers at the point of sale [36].

In a similar manner, the cost-affordability factor also plays an influencing role on physical-activity participation in Nunavut. According to participants, hunting equipment is not affordable and accessible due to its high cost, resulting in fewer hunting activities. Moreover, the exorbitant cost of sporting equipment and registration fees at fitness centers limit Inuit participation in physical activity. Participants’ views on barriers and enablers and attitude toward participation are well corroborated by the results of a previous study that reported cost and affordability as limiting factors to participation in physical activity among Australian Indigenous adults [37]. The prohibitive cost of sporting equipment, expensive gym registration fees, and low social and economic status all combine to form a formidable barrier to physical activity in these Indigenous populations. Similarly, two other studies in Canadian [38] and Australian [39] Indigenous populations reported a lack of access to low-cost recreation activities as a barrier to physical-activity participation.

Apart from direct personal costs in the face of limited income, a majority of Inuit participants in the current study raised concerns regarding inadequate community resources and infrastructural development compared to southern cities and communities. They attributed their low participation in physical activity to very limited availability of community resources, including sporting centers, limited options for traditional and western-oriented sporting activities, and poor conditions of roads and walkways for walking. In addition, absence of road and rail transportation presents a critical infrastructural deficit in Nunavut that adversely affects healthy food supplies from the southern part of Canada, where over 80% of the food items are sourced. In contrast to other jurisdictions in Canada, there are no roads or railways connecting Nunavut to any of the southern provinces, or between any two Nunavut communities. Air travel is the only means of travel and transportation of goods between communities, and from the rest of Canada [40], except during summer when sealift activities take place, and dry and non-perishable items such as canned foods are shipped by sea. This results in a high cost of living: high expenditures on airfares, medical and food supplies, and housing [40].

Participants also raised climate and environmental concerns as barriers to their healthy dietary choices and physical-activity participation. There is a widespread belief amongst Inuit that global warming has disrupted Inuit cultural practices generally, and specifically has adversely impacted their ability to go on the land for caribou hunting or on the sea for seal hunting. Hunting activities have been significantly reduced as a result of the global increase in temperatures, which is melting ice and disrupting the migratory patterns of animals in the Arctic. A study by Wesche and Chan [41] described the moderating role of climate change in the physical environment. The changing climate influences food-species availability and migratory patterns, and indirectly influences the hunter-gatherer movements and diet selection [41]. Additionally, the built environment is underpinned by attributes that influence physical-activity behavior [26,42,43]. Changes to the built environment may alter physical activity participation with attendant health effects. In Nunavut, such changes are an integral part of the social, cultural, and environmental shifts that the Canadian Arctic has experienced over the past five decades [6], and are correlated with decreased physical-activity participation, according to research evidence [26]. For example, subsistence activities (hunting, fishing, and traditional food processing) that were associated with healthy diets and walking behavior have remarkably decreased [6].

Further, participants in the current study indicated that they are likely to follow choices that are perceived “acceptable” based on sociocultural expectations. In clear terms, they expressed how family members and friends play key roles in their dietary choices and influence their participation rates in physical activity. Social networks of families, friends, neighbors, and others have been shown to have remarkable influence on health behaviors [44,45,46] and an individual’s ability to modify unhealthy behaviors [47].

Low participation rates in physical activity are attributed to engagement in substance-use behaviors, and invariably link low energy, interest, and tiredness to substance use. Generally, cigarette smokers spend considerably less time on sports and aerobic activities compared to non-smokers, given that nicotine-dependent smokers are less likely to engage in physical activity [48]. This is important, particularly in Nunavut, where the smoking rate of 63% [49] is at least three times the Canadian average. A recent report by Nunavut’s chief medical officer indicated that the prevalence rate in some Nunavut communities is 84% [50], which is very concerning. When the financial cost of smoking combines with that of excessive alcohol consumption and drug use, the impact is beyond the individual level, as the menace aggravates food insecurity in households and reduces the chances that healthy dietary options will be pursued. Taken together, these findings suggest that while addressing physical inactivity and unhealthy diets in the Inuit population, it is critical to consider a cluster of related unhealthy behaviors for targeted interventions to bolster the chances of success, effectiveness, and long-term sustainability of such efforts. Overall, women readily expressed interest in the research topics, with greater participation rates than men. Moreover, women provided deeper insights than men, describing the various barriers impacting the dietary and physical activity behaviors of members of their households. The women’s passion and depth of insights may be associated with their role as main economic providers and caregivers in most households in the community.

### Study Limitations

The generalizability of the study may be limited due to the complex environment in Nunavut and variabilities across the 25 communities. Community resources, community-level capacities, and infrastructural development vary from one community to another. This particular study was conducted in Iqaluit, the territorial capital and the most diverse, resourced, and populated community in Nunavut. Despite the differences across communities, it would be logical to contextualize our findings, extrapolate, and make some inferences, based on our understanding of Nunavut communities and their characteristics. For example, the barriers identified by participants in Iqaluit may be aggravated in other smaller communities because of the relatively limited resources and community infrastructure. Another limitation is the use of photographs that were provided by the researchers, instead of a more empowering approach in which participants would take and select their own pictures for interviews, as is the case in Photovoice method [51,52]. While the latter might be consistent with research expectations in Indigenous populations and other marginalized groups, we intended to keep the focus of the study on the barriers and enablers. Thus, we confined the area of research interest and discussions by identifying only relevant photographs and utilizing them to prompt interviewees during discussions. This was an “ice-breaker” activity that took place during participant interviews.

It is also worthy to note that only participants who spoke and understood the English language were recruited and included in the study. This presents a limitation, given that unilingual participants were excluded from the study and their contributions might have enriched the data. While there is a concern that our approach may have introduced some bias in the research, we acknowledge that a significant proportion of Nunavut Inuit is fluent in English language, thus providing a good pool of potential participants to provide Inuit perspectives on the subject. This pool covers a broad range of demographic groups independent of age, gender, socioeconomic class, and geographic locations in the community. One comparative advantage of the recruitment of English-speaking participants is that it removed the potential challenges of exorbitant cost and technical difficulties that are usually experienced with translations and reverse translations from and to the local Inuit language (Inuktituk) and the multiple dialects that are spoken by residents who moved from other communities to Iqaluit.

## 5. Conclusions

Despite the limitations to the present exploratory qualitative study, it is evident from our findings that the following key factors are critical to the promotion of healthy energy balance-related behaviors in Iqaluit: cost and affordability of healthy choices; availability of traditional foods and activities; weather conditions and climate change; infrastructure and community resources; social networks of family and friends; and effects of substance use. While the findings of this study may be generalizable across Nunavut communities, additional qualitative studies involving smaller communities across Nunavut are required to deepen our understanding of the subject. Quantitative research across Nunavut communities also is required to assess the relative importance of identified facilitators and barriers in promoting physical activity and healthy eating in the population. These two complementary approaches will further strengthen the development of sustainable intervention efforts. More importantly, the strategic role of transportation infrastructure in the food systems, with regard to access, affordability, and the overall impact on the health and general well-being of Nunavut Inuit, compels a call for both political and policy interventions by the territorial and federal governments. Such interventions would address this critical infrastructural gap that exists between Nunavut and the rest of Canada. In addition, an effective and sustainable food policy intervention by both governments is required to reorient food-supply systems and improve the food environment for the residents.

## Data Availability

Sharing of the qualitative data from this study is restricted due to ethical issues. Confidentiality of Inuit research participants must be protected.
